# Intercostal Artery Aneurysmosis Secondary to Chronic Cocaine Abuse

**DOI:** 10.7759/cureus.21487

**Published:** 2022-01-22

**Authors:** Amit Agarwal, Vishal Kukkar, Kanupriya Vijay

**Affiliations:** 1 Radiology, Mayo Clinic, Jacksonville, USA; 2 Radiology, University of Texas Southwestern Medical Center, Dallas, USA

**Keywords:** abuse, artery, cocaine, aneurysm, intercostal

## Abstract

Intercostal artery aneurysms are rare entities usually seen in connective tissue disorders and inflammatory conditions and syndromes like Ehlers-Danlos syndrome, Kawasaki’s disease, and neurofibromatosis. Spontaneous development of intercostal aneurysm is rare and the presence of multiple aneurysms/aneurysmosis is exceedingly rare. Although there have been a few case reports on aortic aneurysm, coronary artery aneurysms and many on ruptured cerebral aneurysms, we could not find a single case of spontaneous intercostal artery aneurysm secondary to chronic cocaine abuse. We report an exceedingly rare case of intercostal artery aneurysmosis presumably secondary to long-term cocaine abuse. Intercostal artery aneurysm is the least common visceral aneurysm and given the very limited literature on this subject, the pathogenesis is poorly understood.

## Introduction

Intercostal artery aneurysms are rare entities usually seen in connective tissue disorders, inflammatory conditions, and syndromes like Ehlers-Danlos syndrome, Kawasaki’s disease, and neurofibromatosis. Single aneurysms/pseudoaneurysms have occasionally been described in the literature, usually secondary to prior trauma or surgery. Spontaneous development of intercostal aneurysms is rare, and the presence of multiple aneurysms/aneurysmosis is exceedingly rare [1]. Cocaine abuse can lead to various vascular complications ranging from vasospasm, arterial dissection, predisposition to aneurysm formation, and aneurysmal rupture. Few case reports on aortic aneurysms, coronary artery aneurysms and many on ruptured cerebral aneurysms have been published. However, we could not find a single case of spontaneous intercostal artery aneurysm secondary to chronic cocaine abuse [2]. We describe a rare case of multiple bilateral intercostal artery aneurysms in a 42-year-old patient with chronic cocaine abuse and no other underlying etiology.

## Case presentation

**Figure 1:**
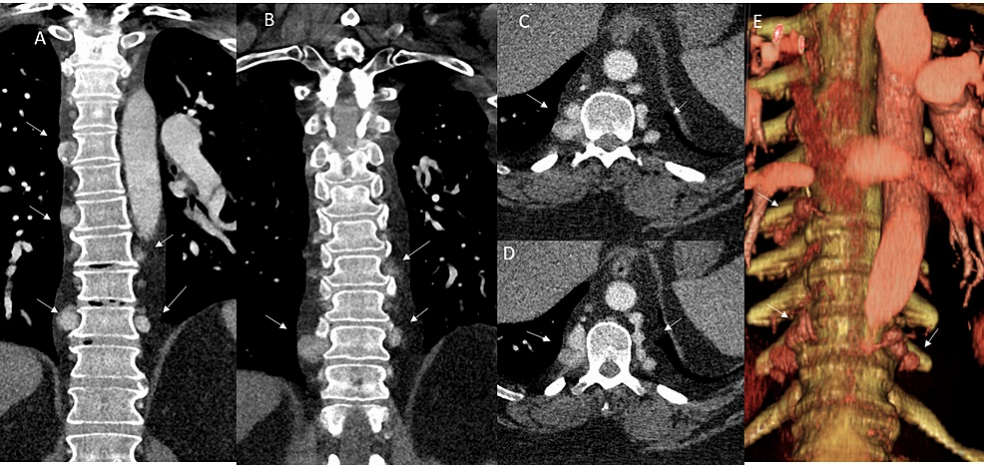
Intercostal artery aneurysmosis on CT angiogram Multiple coronal (A, B) and axial (C, D) CT angiogram images reveal multiple variable-sized intercostal artery aneurysms in the bilateral paravertebral region from the fifth to the tenth (T5-T10) vertebral levels, measuring 0.4-1.7 cm in diameter (arrows). The intercostal origin of these aneurysms was confirmed on the source angiogram and colored volume-rendered image (E).

A 42-year-old male with a history of systolic chronic heart failure (left ventricular ejection fraction 20%) in the settings of cocaine use non-ischemic cardiomyopathy and pulmonary hypertension presented with shortness of breath (SOB). The SOB was described as intermittent, worsened with exertion, and alleviated with rest, oxygen, and sitting up. The patient had been on home bilevel positive airway pressure (BiPAP) therapy with an O_2_ concentrator for many months with gradual worsening of SOB. There was no significant family history, and clinical work-up for collagen vascular diseases was negative. Chest CT angiogram performed to rule out pulmonary thromboembolism (PTE) revealed dilated left ventricle with findings of pulmonary hypertension with no evidence of PTE and no evidence of aortic coarctation. The study also revealed multiple (at least 12) intercostal artery aneurysms in the bilateral paravertebral region from the fifth to the tenth (T5-T10) vertebral levels, measuring 0.4-1.7 cm in diameter (Figures 1A-1D). The intercostal origin of these aneurysms was confirmed on the source angiogram and colored volume-rendered images (Figure 1E).

**Figure 2:**
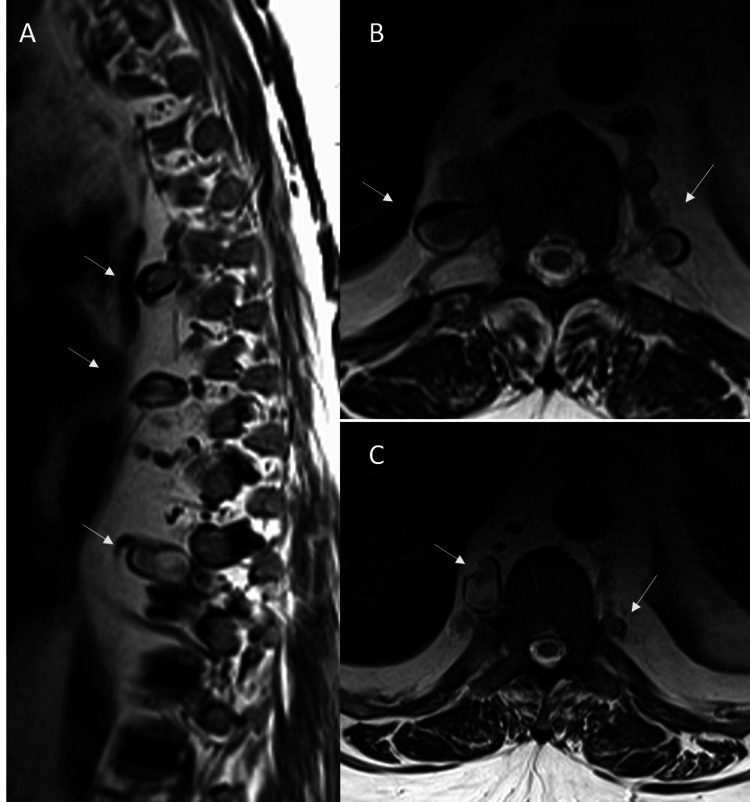
Intercostal artery aneurysmosis on chest MRI Sagittal (A) and axial (B, C) T2-weighted images reveal multiple bilateral intercostal artery aneurysms (arrows) with the standard caliber of the aorta and no evidence of any vascular malformation within the spinal canal.

Further evaluation with MRI of the chest confirmed these findings and ruled out any aortic pathology and any lesion within the spinal canal (Figure 2). Worsening respiratory and cardiac status prevented further work of coronary and brain vasculature. The patient had multiple psychiatric issues, including depression and anxiety and long-term (many years) substance (cocaine) abuse with chemical dependency. He had undergone multiple sessions with a mental health counselor with little benefit. In addition, blood cultures were negative for fungal elements or atypical bacteria. The patient was diagnosed with acute hypoxic respiratory failure secondary to acute on chronic systolic heart failure. Despite extensive therapy and management in the medical ICU (MICU), the patient eventually succumbed secondary to unresolved heart and respiratory failure. Given the established diagnosis of cocaine-induced non-ischemic cardiomyopathy and absence of any additional attributable etiology, the intercostal aneurysms were presumed to be secondary to long-term cocaine abuse. Autopsy was not offered by the clinical team given the adequate medical history and was not requested by the family.

## Discussion

Aneurysmosis of the visceral arteries is usually seen in conditions with connective tissue disorders like Ehlers-Danlos syndrome, Kawasaki’s disease, and neurofibromatosis, secondary to the mesodermal dysplasia of the small arteries in these conditions with weakened walls. It usually involves the mesenteric, hepatic, splenic, and gastro-duodenal arteries. Intercostal aneurysms are reported only in a few cases, and the majority of these are in conjunction with these syndromes and other connective tissue disorders. Few cases of multiple intercostal aneurysms have also been reported in the literature with these conditions and other entities like coarctation of the aorta. It is essential to demarcate these from pseudoaneurysm, which is usually secondary to trauma or iatrogenic and usually seen at one level [1]. The first case of spontaneous intercostal artery aneurysm without any underlying identifiable cause was reported in 2004 by Töpel et al., with a single aneurysm identified [3]. The first case of multiple aneurysms without any underlying cause was first reported in 2013 by Carr et al. with seven bilateral aneurysms that were embolized and excised [1]. Multiple aneurysms in the intercostal arteries have also been reported with coarctation; however, they usually involve the upper thoracic arteries, and the clinical and imaging findings of co-coarctation are very evident.

Mycotic aneurysms in the intercostal region have occasionally been reported; however, they are usually at a single level and are technically pseudoaneurysms rather than true aneurysms. The blood cultures in these patients are usually positive for underlying infections, including atypical bacteria or fungal elements [4]. Intercostal artery aneurysms/pseudoaneurysms can occasionally rupture, resulting in hemothorax with acute chest pain or back pain requiring urgent surgical intervention. Unruptured intercostal aneurysms are usually asymptomatic, with the risk of rupture and hemorrhage directly related to their size [5,6]. Mycotic aneurysms, in general, have a higher tendency for rupture. Given the fact that there is a limited number of cases reported in the literature, there are no clear guidelines for treating these unruptured aneurysms. Endovascular treatment has only been attempted in a minimal number of patients, limiting the credibility of these studies [7,8].

Cocaine abuse has been associated with many cardiovascular complications ranging from myocardial infarction, dilated cardiomyopathy to arterial dissection, vasospasm, and arterial dissection. Cocaine use is also associated with predisposition to aneurysm formation and aneurysmal rupture, including a higher incidence of coronary artery aneurysms. Cocaine-induced cardiovascular complication could be acute like vasospasm, thrombosis and arrhythmias, or could be chronic like cardiomyopathies or early onset of atherosclerosis. There is a direct correlation between the length and amount of cocaine used. There is a multifactorial pathophysiological mechanism primarily through stimulation of adrenergic receptors, increased prothrombotic factors and endothelial cell dysfunction. The average age of subjects with cocaine-induced cardiomyopathy is much younger when compared to other causes of ischemic or non-ischemic cardiomyopathies. Complications like arterial dissection and predisposition to aneurysm formation have the exact underlying pathogenesis of a significant increase in systemic arterial pressure induced by cocaine [9-11]. Although the pathogenesis is still poorly understood, many cases of cocaine-induced coronary artery aneurysms have been reported. The most notable was the study by Satran et al. where it was observed that aneurysms occurred in 30.4% of cocaine users compared with 7.6% of non-users, suggesting a clear link with drug abuse [11]. It has also been postulated that the development of coronary artery aneurysms may be one of the many contributing factors to myocardial infarction. Cocaine remains the most encountered illegal substance in the United States and the most common cause for emergency room visits secondary to drug abuse. Cocaine abuse results in severe episodic hypertension, vasospasm, and subsequent damage to the endothelium. The extremely high incidence of coronary artery aneurysms in cocaine users compared to the cohorts is a strong indicator of a correlation [9]. Our patient had a known diagnosis of cocaine-induced non-ischemic cardiomyopathy. In these settings and in the absence of any other identifiable cause, the intercostal aneurysmosis was presumably secondary to chronic cocaine abuse. Presently, the literature on intercostal artery aneurysms is minimal, and we anticipate these to be more commonly reported in the future, given the increased recognition and advent of multi-detector CT imaging.

## Conclusions

We report an exceedingly rare case of intercostal artery aneurysmosis presumably secondary to long-term cocaine abuse. Chronic cocaine abuse has been associated with numerous cardiovascular complications including arterial dissection and aneurysm formation. Intercostal artery aneurysm is the least common visceral aneurysm, and given the minimal literature on this subject, the pathogenesis is poorly understood. We anticipate that this report will serve as a unique reference for any future condition documentation.
